# Evaluation of the Impact of a Mass Media Campaign on Periodontal Knowledge among Iranian Adults: A Three-Month Follow-Up

**DOI:** 10.1371/journal.pone.0169668

**Published:** 2017-01-06

**Authors:** Mahdia Gholami, Afsaneh Pakdaman, Ali Montazeri, Jorma I. Virtanen

**Affiliations:** 1 Department of Community Oral Health, School of Dentistry, Tehran University of Medical Sciences, Tehran, Iran; 2 Mental Health Research Group, Health Metrics Research Centre, Iranian Institute for Health Sciences Research, ACECR, Tehran, Iran; 3 Research Unit of Oral Health Sciences, University of Oulu, Oulu, Finland; 4 Medical Research Center, Oulu University Hospital, Oulu, Finland; University of Washington, UNITED STATES

## Abstract

**Objectives:**

This study aimed to evaluate the impact of a national media campaign to promote oral health and periodontal knowledge among adults after a three-month follow-up.

**Methods:**

We conducted a population-based study of adults aged 18 to 50 years using a stratified multi-stage sampling method in Tehran, Iran in 2011. The campaign included an animation clip about periodontal health and disease telecast on national TV for ten consecutive days. We used an instrument to assess the effect of the campaign at baseline, immediately after the campaign and after a three-month follow-up. A total of 543 participants responded at baseline and immediately after the intervention, and 294 were interviewed at the three-month follow-up assessment (response rate: 54.1%). We assessed each participant’s periodontal knowledge score, calculated as a sum of correct answers, and the change in their score following the campaign. We then used a five-item questionnaire to evaluate the participants’ opinion of the success of the campaign. We used descriptive statistics and generalised estimating equations (GEE) analysis to conduct the statistical analysis.

**Results:**

The mean score for knowledge improvement from baseline to immediate post-intervention evaluation was higher among those who saw the campaign (0.61) than among those who did not (0.29); the corresponding figures from immediate evaluation to three-month follow-up were -0.17 and 0.15, respectively. Adjusting for baseline values, the GEE analysis demonstrated that improvement in the mean score of post-campaign knowledge associated significantly with age, education and seeing the campaign. Significant interaction between the time since seeing the campaign and whether the participant saw it (p < 0.001) revealed that the mean difference in the knowledge score between the groups who did and did not see the campaign was 0.4 at the immediate evaluation and -0.04 at the three-month follow-up. The participants received the campaign well in terms of being appealing (91.4%), worth bearing in mind (83.4%) and containing valuable information (80.3%).

**Conclusions:**

Our findings indicate that a population-based media campaign promoting oral health and periodontal knowledge among adults had a positive short-term impact, although the effect seemed to plateau after three months.

## Introduction

Patient education can facilitate the promotion of oral health through community awareness campaigns relating to causes, risk factors, symptoms of oral diseases and the importance of preventive practice [1]. Focusing on highly prevalent oral diseases such as periodontitis can be considered a priority, as these inflammatory disorders, which pose a major public health problem in many countries, can, if left untreated, lead to lower quality of life and tooth loss among patients [2].

Health promotion programmes use several theoretical frameworks and models to facilitate changes in behaviour. A new framework known as the behaviour change wheel (BCW) has provided a reliable and useful approach to health topics such as tobacco cessation and obesity reduction [3]. This framework comprises three layers: a “behaviour system”, “intervention functions” and “policy categories”. The behaviour system, located at the centre of the wheel, presents essential conditions of capability, opportunity and motivation to understand behaviour. Intervention functions surrounding the inner layer serve to eliminate the defects of the condition and are supported by the outer layer of policies. In line with this framework, knowledge-based interventions seeking behaviour change would probably fit best under the capability component, which includes possessing the necessary knowledge and skills to change behaviour.

Large proportions of populations can be exposed to oral health messages through various forms of mass media, including electronic media (television and radio), outdoor media (billboards and posters), print media (magazines and newspapers), and digital media (CD, DVD, etc.) or the use of new technologies such as the internet and mobile phones. In many countries the majority of people receive information passively from mass media routes such as television, which can serve as an appropriate tool for the general population as the message can reach large audiences [4,5]. Television reportedly has a positive impact on health attitudes. For instance, some have argued that individuals who receive health information through TV programmes are more knowledgeable of health-related issues than those who do not [6]. Furthermore, evidence has shown that mass media interventions have a more positive impact in developing countries than in developed countries [7].

A review of the impact of mass media on oral health promotion claimed a minor change in behaviour, but acknowledged short-term knowledge improvement [8]. However, the review was inconclusive due to the small number of studies it included and its methodological limitations. Other studies have shown mass media to be an important tool in promoting public knowledge of oral health, shifting attitudes and encouraging changes in behaviour [9–12]. Although several previous studies have shown improvement in oral health knowledge in post-campaign surveys [5,10,12,13], the evidence needed to assess the sustained positive impact of the campaign over short-, medium- or long-term follow-ups is insufficient. Overall, few follow-up assessments have evaluated the impact of mass media on oral health [13,14]. In some studies, the campaigns featured different components, including mass media and supplementary oral health education by dental staff and teachers [13,14]. Evidence of the sustainability of mass media campaigns in relation to campaigns promoting oral health at the population level, particularly in developing countries, is limited and inadequate.

Periodontal disease is a major public health problem in Iran that places a heavy annual cost burden on the health care system. We therefore launched a mass media campaign and evaluated the participants’ knowledge of periodontal health immediately and three months after viewing the campaign. The aim was to investigate whether a mass media campaign focused on oral health education can improve participants’ knowledge of periodontal disease as a predisposing factor for healthy behaviour. This paper reports on the follow-up data of the campaign. We found that periodontal knowledge among adults improved immediately after the campaign [5]; however, this report focuses on the three-month follow-up data, which has not been previously reported due to the unavailability of the data at the time of writing of the first report. Besides, including the three-month follow-up data in the first report could confuse the reader. Consequently, this paper aimed to focus on the sustainability of a national media campaign promoting oral health and periodontal knowledge among adults in Tehran after a three-month follow-up.

## Methods

### Study design and population

We conducted a population-based intervention study among working-age adults (18–50 years) in Tehran, Iran in 2011. We used a stratified multi-stage sampling method to select the participants from all 22 districts of Tehran. Details of the sampling have been reported previously [5].

**Fig 1:**
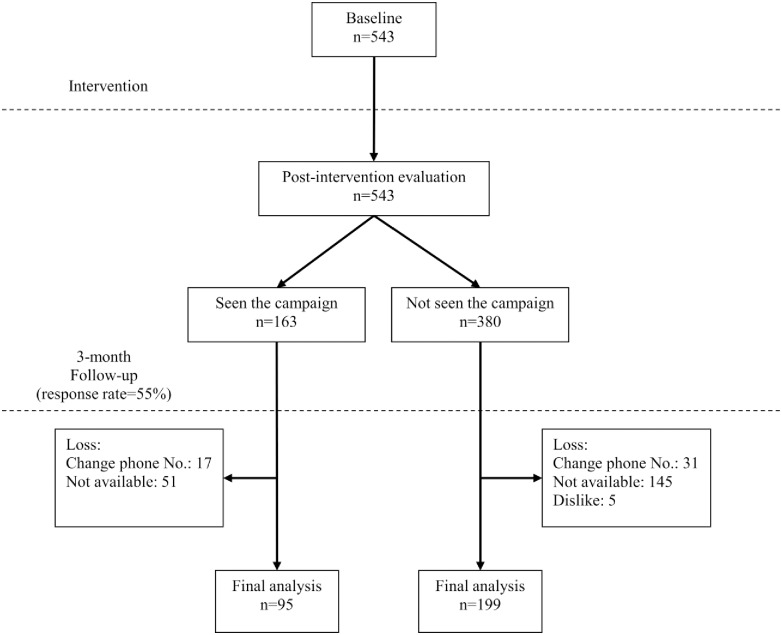
Flow chart of study participants.

A team of trained interviewers collected the data identically at three phases: baseline (before launching the campaign), post-intervention (immediately after the campaign) and follow-up (three months after the post-intervention evaluation). A flow chart of the study demonstrated that 543 individuals participated in the baseline evaluation and in the immediate post-intervention evaluation. Of these, 294 participants responded to the questionnaire at the three-month follow-up (Fig 1).

### Educational intervention and assessment

The intervention was planned as a national TV campaign that featured an animation clip about periodontal health and disease; the campaign was telecast for ten consecutive days followed one week later by a TV message reminder for seven days. At the time of the study, the capital city of Tehran had five national and one local TV channel. The clip was telecast nationwide on four national channels and on one local TV channel in the capital. The animation clip was about a conversation between a young couple before their wedding ceremony. The groom was worried about some symptoms that he had in his mouth, including red gingiva, gum bleeding and halitosis. The bride, who was a dental student, explained that those symptoms are the early common signs of periodontal disease and can be avoided through preventive measures such as oral hygiene and smoking cessation. Furthermore, she mentions the major role played by dental plaque in the aetiology of the disease.

We used a questionnaire to assess the participants’ periodontal knowledge before the campaign, immediately after the campaign, and at the three-month follow-up. The valid and reliable ten-item questionnaire was developed to assess the participants’ baseline periodontal knowledge [15]. We calculated and assessed the face and content validity of the instrument [scale content validity index (0.92), content validity ratio (0.87)] as acceptable, and report the reliability as a percentage of test-retest agreement, which exceeded 70%. For internal consistency, we calculated a Cronbach’s alpha of 67%. The questions related to different aspects of periodontal disease, including gingival appearance, causes and outcomes of periodontal disease, and the impact of systemic disease on periodontal health. Of these, three questions enquired about periodontal health issues presented in the animation clip. We derived these three questions from our previously validated instrument to assess the impact of the campaign on periodontal knowledge. The multiple-choice questions were related to the definition of dental plaque (response alternatives: ‘Soft, colourless and sticky deposits on the teeth that contain microbial and food debris’, ‘Black or brown stains from food or drinks on the teeth’, ‘It is the same as dental calculus, which contains microbial and food debris’, ‘A hard, coloured mineral layer on the inner surface of the teeth’ and ‘Don’t know’), the cause of gum disease (response alternatives: ‘Oral aphtus’, ‘Dental plaque’, ‘Excessive use of antibiotics’, ‘High sugar consumption’ and ‘Don’t know’) and the early signs of the disease (response alternatives: ‘Tooth discoloration’, ‘Tooth mobility’, ‘Tooth abrasion’, ‘Red gingiva’ and ‘Don’t know’).

A few additional questions also enquired about the respondents’ background, including their age, education, marital status, employment and economic status. We determined the participants’ economic status based on the living area of their apartment/house (in square metres per person). We used this proxy measure because previous studies in Iran have shown it to be one of the best measures of economic status; because Iranians often work at more than one job, income information is often unreliable [16,17]. We subsequently defined three groups based on the distribution of the data: group I) < 23.8 m2/p, group II) 23.8–36.7 m2/p and group III) ≥ 36.7 m2/p); group I represents the least affluent of the groups [5].

To evaluate health education programmes, we used some indicators of success and performance derived from standard questions developed by the US Office of the Cancer Communication Health Testing Service regarding the pretesting evaluation of a public mass media campaign [18]. These questions have also been recommended for use after the implementation of media campaigns [18]. We selected the indicators so as to assess the success of the campaign from the perspective of those participants who had seen it and according to the following five criteria: “appealing”, “provides valuable information”, “relevant to the audience”, “worth bearing in mind” and “contains information worth recommending to others”, reported as a percentage value.

In the present study, we evaluated the impact of the periodontal campaign on the participants’ level of knowledge immediately after the intervention and after the three-month follow-up, and then compared the findings to their baseline periodontal knowledge. For this purpose, each question answered correctly was scored “1”, and incorrect answers, “0”. We calculated each participant’s total score for periodontal knowledge (range 0–3) by adding up the number of correct answers. We evaluated the difference between knowledge scores from baseline to post-campaign evaluation as improvement in periodontal knowledge.

### Statistical analysis

We used SPSS package version 23 (SPSS Inc., Chicago IL, USA) to analyse the data. At the three-month follow-up, and with a response rate of 54.1%, we tested for patterns in the missing data [19,20]. To this end, we used the Chi-square test to compare the characteristics of the participants and non-participants at the three-month follow-up (Table 1). Because we detected no statistically significant differences between the groups, any patterns in the missing data were considered Missing Completely At Random (MCAR) (i.e., individuals with missing data at the follow-up were randomly scattered throughout the sample). We therefore conducted the analysis using available cases without fear of bias in the findings [21].

**Table 1:** Characteristics of the study participants in baseline and those who participated and lost in three-month follow-up.

	Baseline	Three-month follow-up	Lost sample	P*
	N = 543	N = 294	N = 249	
	No. (%)	No. (%)	No. (%)	
**Gender**				0.59
Male	279 (51.4)	148 (50.3)	131 (52.6)	
Female	264 (48.6)	146 (49.7)	118 (47.4)	
Total	543 (100)	294 (100)	249 (100)	
**Age group**				0.50
18–24	143 (26.3)	73 (24.8)	70 (28.1)	
25–34	178 (32.8)	97 (33)	81 (32.5)	
35–44	128 (23.6)	76 (25.9)	52 (20.9)	
> 45	94 (17.3)	48 (16.3)	46 (18.5)	
Total	543 (100)	294 (100)	249 (100)	
**Education**				0.01
Primary/Illiterate^1^	91 (16.8)	38 (13)	53 (21.3)	
High school	229 (42.3)	122 (41.8)	107 (43)	
University	221 (40.9)	132 (45.2)	89 (35.7)	
Total	541 (100)	292 (100)	249 (100)	
**Marital status**				0.39
Married	183 (34.1)	95 (32.5)	88 (35.8)	
Single	355 (65.9)	197 (67.5)	158 (64.2)	
Total	538 (100)	292 (100)	246 (100)	
**Employment**				0.92
Employed	270 (50.3)	145 (50.1)	125 (50.6)	
Unemployed	41 (7.6)	21 (7.2)	20 (8.1)	
Student	64 (11.9)	33 (11.4)	31 (12.6)	
Housewife	152 (28.3)	85 (29.3)	67 (27.1)	
Other	10 (1.9)	6 (2)	4 (1.6)	
Total	537 (100)	290 (100)	247 (100)	
**Economic status**^2^				0.57
Low	164 (34.7)	78 (30)	65 (30.7)	
Moderate	143 (30.3)	86 (33.1)	78 (36.8)	
High	165 (35.0)	96 (36.9)	69 (32.5)	
Total	472 (100)	260 (100)	212 (100)	

^1^ Primary/Illiterate: less than nine years of education

^2^ derived from average living area in square meter per person (m^2^/p)

*Chi-square, p<0.05

To assess the effect of variables such as the baseline knowledge score, time after viewing the campaign, viewing the campaign and socio-demographic variables on the mean score for post-campaign knowledge we conducted a generalised estimating equation (GEE) analysis. While the GEE analysis revealed significant interaction between the two main factors of time after viewing the campaign and group (viewing the campaign or not), we computed the estimated mean difference in post-campaign knowledge scores between the participants who had viewed the campaign and those who had not separately at each measurement point [22].

### Ethics statement

The Tehran University of Medical Sciences and Health Services supported the study through grant no. 90-01-69-12892. The interviewers, who received special training to explain the study objectives to the participants and to ask for their permission, interviewed those who consented to participate in the study, including illiterate subjects who provided their verbal consent. The interviewers presented the questions to the respondents and recorded their answers on forms. The questionnaire was not self-administered. It was coded, and answers were recorded anonymously. Participants were informed that their information would remain confidential and that they were free to withdraw from the study at any time. The ethics committee of the Tehran University of Medical Sciences (code no. 90-01-69-12892) approved the study.

## Results

The baseline socio-demographic characteristics of the respondents who participated, but were lost at the three-month follow-up, appear in Table 1. The participants and non-participants showed no significant differences in their characteristics.

Responses to questions about periodontal knowledge after viewing the campaign or not appear in Table 2. The percentage of correct answers among all the participants generally increased from baseline to immediate post-intervention evaluation. However, this improvement was greater among those respondents who saw the campaign than among those who did not see it. In the group who viewed the campaign, the highest and lowest levels of improvement in correct answers associated with the questions ‘early signs of gum disease’ (27.3%) and ‘the definition of dental plaque’ (15.6%), respectively. In this group, the percentage of correct answers for the question ‘early signs of gum disease’ increased from the immediate post-campaign evaluation to the three-month follow-up. For the other two questions (‘the definition of dental plaque’ and ‘causes of gum disease’), the corresponding figures decreased, but the figures remained higher than at baseline.

**Table 2:** Total number of the subjects (%) reported the correct answers to the knowledge questions at pre- and post-campaign evaluations.

Questions	Baseline*		Post-intervention		Follow-up	
	Seen	Not seen	Seen	Not seen	Seen	Not seen
	No. (%)	No. (%)	No. (%)	No. (%)	No. (%)	No. (%)
What is dental plaque?^1^	21 (13.0)	35 (9.2)	46 (28.6)	45 (11.9)	18 (18.9)	26 (13.1)
What causes gum disease?^2^	25 (15.4)	37 (9.8)	56 (34.8)	65 (17.2)	22 (23.9)	36 (18.6)
Which one is the early sign of gum disease?^3^	66 (41.0)	155 (41.1)	110 (68.3)	230 (60.8)	62 (68.9)	142 (73.2)
Total	163 (100)	380 (100)	163 (100)	380 (100)	95 (100)	199 (100)

*According to post-intervention category

^1, 2, 3^ The correct answer:

^1^ Soft, colourless and sticky deposits on the teeth that contain microbial and food debris;

^2^ Dental plaque;

^3^ Red gingival.

**Fig 2:**
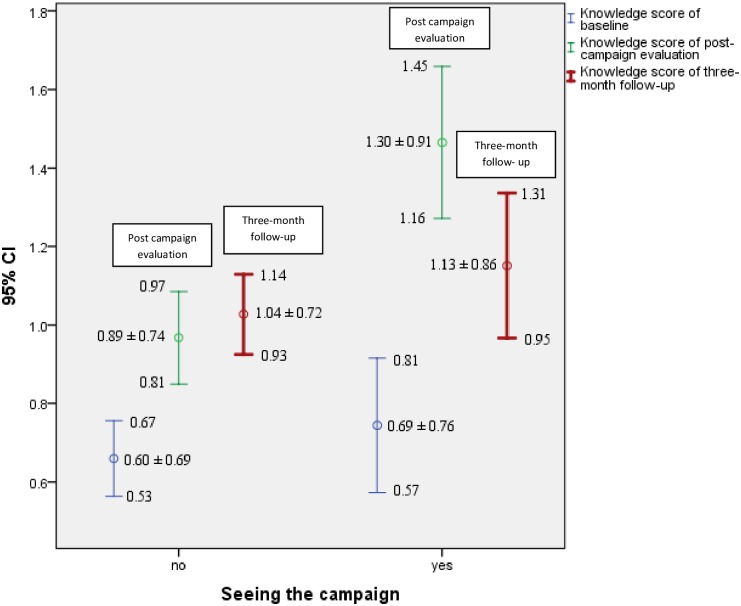
Mean score of knowledge among those “who had seen” and those “who had not seen” the campaign at baseline, immediately after the campaign and at the three-month follow-up.

Fig 2 shows the mean scores for periodontal knowledge in the pre- and post-campaign evaluations of the participants who had viewed the campaign and those who did not. The improvement in knowledge from baseline to immediate post-intervention evaluation was higher among those participants who had viewed the campaign (0.61) than among those who had not (0.29). Between the immediate post-campaign evaluation and the three-month follow-up, the mean knowledge score declined in the group that had seen the campaign (-0.17), although the score was still higher at the three-month follow-up than at baseline, if not statistically significant (p = 0.843).

After adjusting for baseline knowledge values, the GEE analysis revealed a significant relationship between improvement in the mean post-campaign knowledge score and age (25–34 vs. 18–24, p = 0.036), education (high school vs. primary/illiterate, p = 0.001; university vs. primary/illiterate, p < 0.001) and measurement point (p = 0.011). In addition, viewing the campaign (p < 0.001) and the interaction between viewing the campaign and time (p = < 0.001) were associated with improvement in mean score of post-campaign knowledge (Table 3).

**Table 3:** The results of GEE analysis for assessing the effect of independent variables on mean periodontal knowledge scores immediately after the campaign and at three-month follow-up.

Variable	Category	EST.	SE	P*
**Intercept**	-	0.183	0.122	0.134
**Gender**	Male	ref.		
	Female	0.154	0.088	0.083
**Age**	18–24	ref.		
	25–34	0.209	0.099	**0.036**
	35–44	0.162	0.110	0.141
	≥ 45	0.197	0.118	0.097
**Education**	Primary/illiterate	ref.		
	High school	0.289	0.088	**0.001**
	University	0.393	0.103	**<0.001**
**Marital status**	Married	ref.		
	Single	-0.049	0.102	0.632
**Employment**	Employed	ref.		
	Unemployed	-0.143	0.121	0.239
	Student	0.003	0.107	0.974
	Housewife	0.026	0.109	0.812
**Economic status(m**^**2**^**/p)**	-	0.001	0.001	0.309
**Baseline knowledge Score**	-	0.313	0.044	**<0.001**
**Time**	-	0.050	0.019	**0.011**
**Seeing the campaign**	No	ref.		
	Yes	0.400	0.085	**<0.001**
Interaction of time*seeing the campaign	-	-0.148	0.041	**<0.001**

EST. = estimation; SE = standard error

* p<0.05

Because the GEE model, which takes into consideration the two post-campaign evaluations, shows a significant interaction between time and having viewed the campaign, we report the immediate and three-month post-campaign evaluations separately. At the immediate post-campaign evaluation, those participants who had seen the campaign had a higher knowledge score than did those who had not (viewed = 0.583, not viewed = 0.183, mean difference = 0.4). At the three-month follow-up, this mean difference decreased between those who viewed the campaign and those who did not (seen = 0.289, did not see = 0.333, mean difference = -0.04) (Fig 2).

The success of the campaign as a percentage value for each item appears in Table 4. A majority of the respondents who had viewed the campaign found that the campaign was appealing (91.4%) and worth remembering (83.4%). A minority reported recommending the information in the campaign to a family member or other relative (29.4%).

**Table 4:** Frequency of the participants' responses regarding the success of the campaign (n = 163).

Questions	Yes
	No. (%)
In your opinion, was the campaign appealing and interesting?	149 (91.4)
Did the campaign include valuable and effective information regarding gums health?	131 (80.3)
Was the content of the campaign relevant to your condition?	71 (43.5)
Do you think the campaign had value of keeping in the mind?	136 (83.4)
Have you recommended the information of the campaign to your family member or other relatives?	48 (29.4)

## Discussion

This study reports the effect of a mass media campaign to promote oral health and periodontal knowledge among adults in Tehran after a three-month follow-up. Those participants who viewed the campaign generally evaluated its success as high. The periodontal knowledge of those participants who had viewed the campaign, showed significant improvement immediately after the intervention over that of those who had not, although this improvement in knowledge diminished by the three-month follow-up.

A study of five- to seven-year-old Scottish children and their mothers involving a mass media campaign that used television commercials and local magazines as well as a package of dental health material for home use distributed through schools in an effort to raise oral health awareness reported similar findings. This Scottish study reported high retention of oral health knowledge at the immediate post-campaign evaluation, but lower at the one-month follow-up [9]. Rise et al., however, demonstrated improvement in periodontal knowledge among Norwegian adults through a mass media campaign at a two-year follow-up [23].

In our study, those participants aged 25–36 years and those with higher education showed more post-campaign improvements in periodontal knowledge. A similar study among Swedish adults revealed a similarly significant relationship between higher education and greater periodontal knowledge after a mass media campaign [24]. However, that study showed no significant relationship between knowledge and age group. In addition, in our study, those respondents with a higher periodontal knowledge score at baseline demonstrated much greater post-campaign periodontal knowledge. These findings demonstrate that educational level and baseline knowledge of periodontal health are important factors in designing similar media campaigns to promote oral health. These results therefore support the development of clear, simple messages for adults with a lower level of education and baseline knowledge, particularly for the middle-aged. However, we acknowledge that knowledge improved more among the more highly educated subjects, which may lead to a higher risk of inequality in the community. Our results highlight the need to design more specific interventions based on individual characteristics such as education level. This confirms the finding of the systematic review that introduced Motivational Intervention (MI) as the most effective method for changing oral health behaviour [25]. This client-centred approach facilitates the change process based on the client’s personal characteristics by resolving uncertainties and supporting the client’s decisions to promote oral health. In addition, the less-educated are more likely to be a deprived class of society, where oral health is seldom a priority. Thus, they might not follow campaign recommendations precisely.

One strength of our study is that it took place at the population level using a stratified multi-stage sampling method to improve adults’ periodontal knowledge. The information was disseminated through national TV channels in the form of an animation clip in order to be more appealing and effective. We used a valid and reliable instrument to collect data on periodontal knowledge. This was important because studies at the community level may encounter problems, such as difficult accessibility to adults, missing data at multiple follow-ups, and other issues.

Several factors, including how well the viewers received and understood the campaign along with the duration and frequency of their exposure to the message, may influence the effectiveness of mass media campaigns [14]. In our study, we evaluated the campaign’s success in terms of factors such as whether it was worth remembering and offered valuable and appealing content and confirmed that the viewer found it favourable. This result is in line with the findings of a study from Scotland that reported on a successful dental health mass media campaign which the majority of participants considered to be quite good and very good [26].

In our study, the effect of the media campaign diminished with time. The impact of health education is temporary, as Kay and Locker indicated in their review [8]. Previous oral health campaigns extending over longer periods to disseminate a message have also demonstrated more sustainable knowledge improvement: a two-year periodontal campaign among adults in New Zealand, for example, demonstrated improvement in knowledge and periodontal health status up to three years after the end of the campaign [14]. In addition, the use of different types of mass media, such as TV, radio, newspapers, billboards, among others, may reach a larger audience for knowledge improvement [23].

In our study, approximately one third of the respondents had viewed the TV clip, thus demonstrating acceptable audience coverage. Our campaign consisted of a clear message for heterogeneous audiences with different levels of education and expectations. Thus, we were aware that the message might not be equally persuasive for all participants; this variable reception was also evident among Swedish adults exposed to a single message about periodontitis, which led to greater knowledge retention among educated participants [24,4].

The participants who did not see the campaign showed some improvement in periodontal knowledge during the study, but this improvement was not statistically significant and could be related to inter-group contamination, a common phenomenon in similar studies which use mass media to disseminate educational messages to the community. The participants who did not view the campaign may have received the information from their relatives or friends who had viewed the campaign. More research is therefore needed to identify the sustainability of the impact of mass media on the promotion of oral health with no inter-group contamination. On the other hand, knowledge improvement after three months in the group that did not view the campaign could be related to oral health activities (personal self-care, oral health literacy, etc.) at the national level conducted by the Ministry of Health and Medical Education. The transient effect of oral health education efforts, as reported in previous studies [8], underscores the need for recurrent reminding in various formats [4,23,27].

This research took place in Tehran, and the findings of the study are from Iranian adults. This may raise the question of whether a sample taken from the urban capital is actually representative of the rest of the country. Previous studies have shown Tehran to be a vast metropolitan area with a mixture of different cultural, socioeconomic and ethnic backgrounds [28], which supports the idea that a sample taken from the general population in Tehran may represent the urban population of the rest of the country.

The experience of this study confirms that using mass media to promote health is expensive. However, since other means will likely incur the same costs, involving mass media–and especially electronic media–might offer a more effective means to communicate with the general public, of whom many may be illiterate. Evidence also suggests that media interventions effectively provide a common source of information, especially in developing countries [7].

## Conclusion

Our study showed the positive effect of population-based national media campaign to promote short-term oral health and periodontal knowledge among adults, although the effect diminishes with time. Recurrent reminders are therefore necessary to sustain this improvement in knowledge.

## Supporting Information

S1 File A minimal set of data for the study. (SAV)
